# Kombucha as a Reservoir of Probiotic Yeasts Tailored for Plant-Based Fermentations

**DOI:** 10.3390/jof12080559

**Published:** 2026-07-30

**Authors:** Laura Canonico, Alice Agarbati, Federica Zannini, Maurizio Ciani, Francesca Comitini

**Affiliations:** Department of Life and Environmental Sciences, Polytechnic University of Marche, Via Brecce Bianche, 60131 Ancona, Italy; l.canonico@univpm.it (L.C.); a.agarbati@univpm.it (A.A.); f.zannini@pm.univpm.it (F.Z.); m.ciani@univpm.it (M.C.)

**Keywords:** kombucha, probiotic reservoir, yeast characterization, non-*Saccharomyces*, functional fermentation, SCOBY

## Abstract

Kombucha is a reservoir of health-promoting yeast strains. In this study, molecular characterization of the Kombucha microbiota led to the identification of 52 distinct yeast isolates, highlighting a complex community, where *Brettanomyces anomalus* was identified as the dominant yeast species. Other key species were identified, such as *Pichia membranifaciens*, *Torulaspora delbrueckii*, *Zygosaccharomyces bailii*, *Starmerella bacillaris*, and *Meyerozyma guilliermondii*. These isolated yeasts were screened for essential probiotic requirements, demonstrating excellent functional traits showing over 80% survival rate under simulated gastric and gastrointestinal conditions. Regarding the surface properties, wide cell hydrophobicity and auto-aggregation capabilities were seen. Broad antioxidant ability and robust antimicrobial activity against *Escherichia coli* and *Staphylococcus aureus* were observed, highlighting a protective function. Two strains of *T. delbrueckii* and one strain of *Z. bailii* stood out as probiotic candidates and were technologically evaluated in different culture combinations: yeast formulation pools using a specialized “probiotic pool” (PP) and a broader “yeast pool” (YP) both yielded successful analytical and sensory profiles. A strain of *Lachancea thermotolerans*, isolated from a distinct natural matrix, was employed as a technological booster to drive a yeast-based, bacteria-free kombucha. This study confirmed that specialized kombucha yeasts were not just fermentation drivers, but sustainable assets for developing novel functional plant-based probiotic beverages.

## 1. Introduction

Kombucha is a traditional fermented tea that is attracting growing interest due to its potential beneficial effects, including antioxidant, anti-inflammatory, and antimicrobial properties [1,2]. The fermentation process is driven by a Symbiotic Culture of Bacteria and Yeast (SCOBY), which creates a complex biochemical environment through microbial interactions [3,4]. While acetic acid bacteria (AAB), particularly of the genus *Komagataeibacter*, are well-characterized for their role in organic acid production and pellicle formation [5,6], the yeast fraction, often dominated by *Brettanomyces*, *Zygosaccharomyces*, and *Saccharomyces*, remains a vast and unexplored reservoir of microbial diversity that significantly influences the beverage’s functional and sensory profile [3,7,8]. In recent years, the demand for non-dairy probiotic sources has intensified, shifting research focus toward fermented plant-based matrices as delivery vehicles for functional microorganisms [9,10].

The transition toward plant-based diets has exposed a critical need for robust probiotic starters capable of thriving in matrices rich in anti-nutritional factors and complex carbohydrates. While traditional dairy probiotics (e.g., *Lactobacillus* and *Bifidobacterium*) often show poor viability in non-dairy environments, non-conventional yeasts from fermented beverages like kombucha possess innate metabolic plasticity. These yeasts can tolerate the high phenolic content and low pH typical of plant-derived substrates, making them ideal candidates for the development of sustainable functional foods [10,11].

Non-*Saccharomyces* yeasts intrinsic to kombucha, such as *Brettanomyces*, *Zygosaccharomyces*, and *Pichia* genera, exhibit remarkable adaptation to high acidity and osmotic stress [3,12,13]. Unlike conventional bacterial probiotics, these yeasts offer unique clinical advantages: they possess inherent resistance to antibiotics, allowing for administration during antibiotic therapy, and can neutralize bacterial toxins while modulating the host immune response [14,15,16].

Unlike bacterial probiotics, yeasts offer a unique safety profile due to their large cell size, which prevents translocation across the intestinal epithelial barrier in immunocompromised hosts. Moreover, their ability to survive and remain metabolically active in the presence of common antibiotics ensures the maintenance of intestinal homeostasis during pharmacological treatments, a feature that bacterial starters cannot provide [14,15,16].

However, to be classified as probiotics, yeast isolates must survive the harsh conditions of the gastrointestinal (GI) tract, including exposure to low pH and bile salts, and demonstrate functional benefits such as auto-aggregation, biofilm formation, and pathogen inhibition [17]. Beyond basic survival, there is growing interest in specialized functional traits, such as phytase activity. The degradation of phytates, anti-nutritional factors common in plant-based diets, by yeast-derived phytases can significantly enhance the bioavailability of essential minerals like iron, zinc, and calcium, further increasing the nutraceutical value of the final product [11].

The enzymatic repertoire of non-*Saccharomyces* yeasts, particularly their phytase and beta-glucosidase activities, represents a transformative tool for plant-based fermentations. By reducing the concentration of phytic acid, these microorganisms not only enhance the absorption of essential micronutrients but also release bioactive aglycones from phenolic compounds, potentially increasing the antioxidant and anti-inflammatory capacity of the final beverage [11,18]. Furthermore, the metabolic interaction within multi-strain yeast consortia during plant-based fermentations can induce cross-feeding phenomena. This mechanism leads to the active esterification of organic acids and the liberation of volatile organic compounds (VOCs), which significantly modulate the aromatic complexity, shifting the sensory profile away from off-flavors toward desirable floral and fruity notes.

Recent studies suggest that kombucha may harbor strains with superior metabolic versatility compared to those isolated from other fermented foods, yet systematic screening for their multifunctional aptitudes remains limited. Unlike traditional SCOBYs, where AAB prompt aggressive acidification, yeast-only fermentations exhibit slower kinetics, requiring extended incubation to achieve chemical and pH stability. To overcome this sluggish behavior without AAB, *L. thermotolerans* represents a strategic ‘technological booster’. Widely used in winemaking but unexplored in plant-based matrices, *L. thermotolerans* produces high amounts of L-lactic acid from sugars [19]. In a bacteria-free kombucha model, its bio-acidification ensures microbial safety while replacing pungent acetic notes with a smoother fixed acidity, potentially enhancing sensory acceptance. Based on these considerations, this study tests three core hypotheses: (i) non-*Saccharomyces* isolates from kombucha possess unique physiological adaptations, yielding gastrointestinal survival and bio-adhesion rates comparable or superior to commercial probiotic benchmarks; (ii) a select consortium of these robust strains can successfully ferment plant-based matrices without AAB; (iii) co-inoculating this probiotic pool with *L. thermotolerans* (strain DiSVA 322, previously isolated in our laboratory from a natural matrix) [20] creates a controlled bio-acidification system that limits excessive pH drops while enriching the beverage’s aromatic and functional profiles through metabolic synergy.

This research provides a scientific framework for the selection of specialized yeast pools to standardize the production of high-value functional beverages.

## 2. Materials and Methods

### 2.1. Sample Collection and Microbial Enumeration

**Figure 1:**
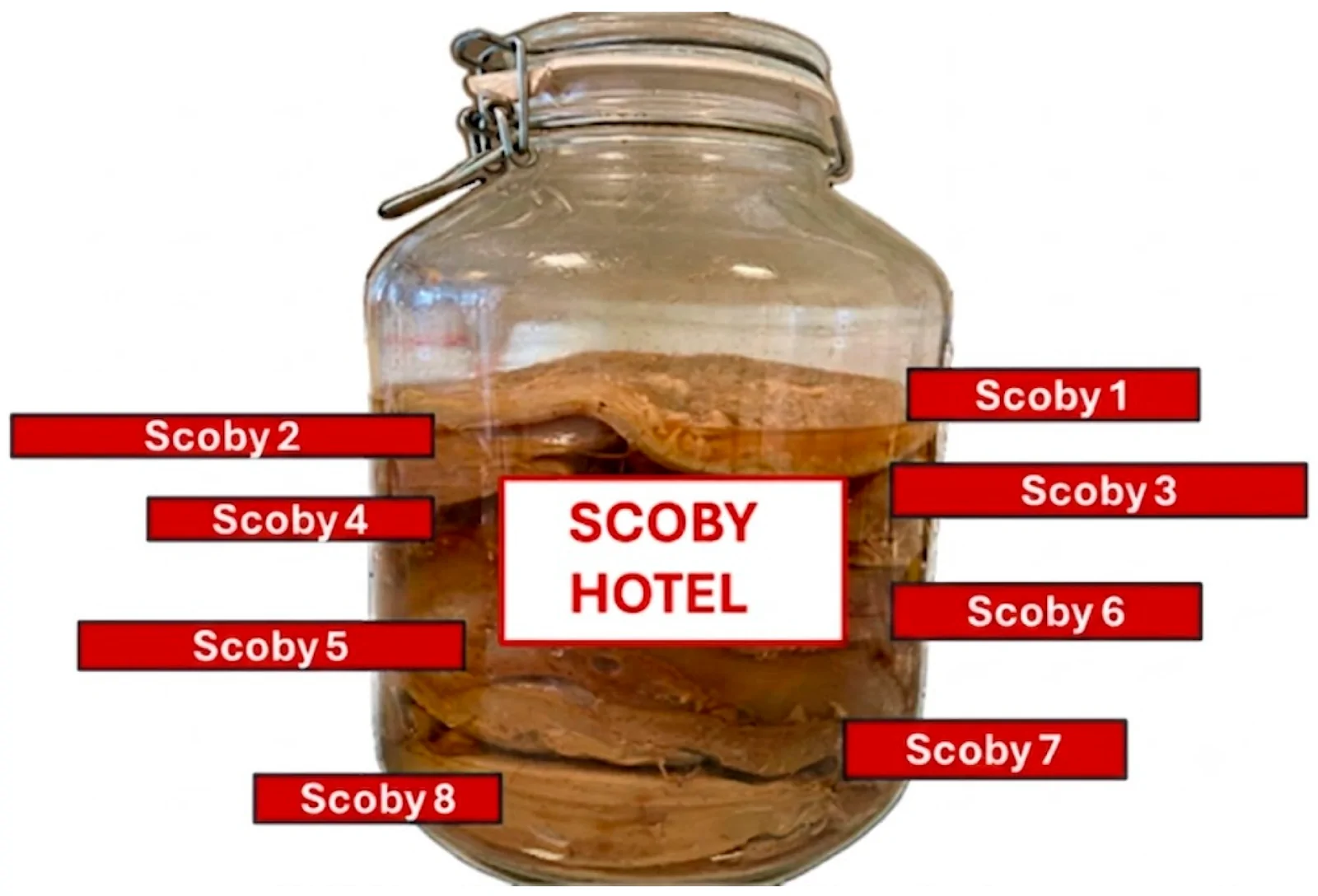
Long-term preservation of kombucha pellicles in a SCOBY Hotel. The labels indicate the different starter cultures used as sources for the isolation of novel probiotic yeast candidates.

A representative “SCOBY Hotel” (Figure 1) from a local non-industrial tea mushroom farm, used for the long-term preservation and maintenance of symbiotic cultures, was used in this study. Individual films (labeled SCOBY 1–8) were maintained in a starter culture (back slop) prior to isolation of the specific yeast strains studied in this research. The SCOBY Hotel contained pellicles at varying maturation stages, ranging from approximately one month (Scoby 1) to one year (Scoby 8). This age gradient allowed for the investigation of yeast microbiota across different long-term storage periods. For microbial analysis, 30 g of each SCOBY was homogenized in sterile peptone water (0.9% *w*/*v*). Serial dilutions were spread-plated onto WL nutrient agar (Oxoid, UK) for the enumeration of the yeasts and incubated at 25 °C for 72–96 h to quantify the yeast and mold populations (log CFU/g), and PCA (Liofhilchem, Roseto degli Abruzzi, Teramo, Italy) was used for mesophilic bacterial growth. Each SCOBY was analyzed using three separated plugs that were harvested.

### 2.2. Isolation and Molecular Identification of Yeast Strains

Following a comprehensive macro- and micro-morphological evaluation based on colony characteristics and optical microscopy, the initial pool of 102 isolates was grouped according to phenotypic similarities. Representative strains from each morphological cluster (n = 58) were selected for genetic sequencing, proportional to the relative frequency of each group within the initial population. Taxonomic identification was achieved by PCR amplification and sequencing of the ITS1-5.8S rRNA-ITS2 region using ITS1/ITS4 universal primers. Briefly, a primer set comprising ITS1 (5′-TCCGTAGGTGAACCTCGCG-3′) and ITS4 (5′-TCCTCCGCTTTATTGATATGC-3′) was used [21]. The resulting PCR products were separated by horizontal electrophoresis (Bio-Rad, Hercules, CA, USA) on a 1.5% (*w*/*v*) agarose gel in 0.5× TBE buffer, stained with ethidium bromide, and visualized under UV light. Each yeast isolate was then identified by sequencing the purified amplicons. Species assignment was performed by comparing the obtained nucleotide sequences with reference sequences available from the NCBI GenBank database using the BLASTn algorithm. Out of the 58 sequenced strains, 52 isolates were successfully identified as yeasts at the species level, exhibiting a query coverage ≥ 98% and a sequence identity percentage ≥ 99% with the closest type strain. To avoid database redundancy, the nucleotide sequences of the 13 representative master strains selected for each identified taxonomic cluster were deposited in the NCBI GenBank data library under accession numbers PX503675 to PX503687. To assess intra-specific genetic diversity and identify unique biotypes, ISSR-PCR fingerprinting and RAPD-PCR with the primer M13 were performed according to standardized protocols [21,22]. The PCR products were separated by electrophoresis on 1.5% (*w*/*v*) agarose gels in 1X TAE buffer at 80 V for 2 h. Gels were stained with ethidium bromide, visualized under UV light, and photographed. The electrophoretic profiles were visually compared; isolates exhibiting identical band patterns were assigned to the same biotype, while profiles with at least one distinct and reproducible band difference were classified as distinct and unique biotypes, leading to the collection of 18 distinct biotypes for further probiotic screening.

### 2.3. Safety Evaluation and Probiotic Characterization

The safety attributes of the 18 yeast biotypes were experimentally evaluated through hemolytic, gelatinase, and DNase enzymatic activity assays to ensure non-pathogenicity prior to functional characterization assays, in accordance with FAO/WHO guidelines [23]. Probiotic potential aptitude was screened while applying the protocols standardized in previous work [24], and gastrointestinal tolerance and survival under simulated gastric (pH 2.0, pepsin 3 g/L) and intestinal (pH 7.0, 0.3% bile salts) conditions at 37 °C were evaluated. Additional features such as surface properties (auto-aggregation in 24 h) and cell surface hydrophobicity (hexadecane partition) were also assayed. Adhesion assays using human Caco-2 cells and biofilm formation were tested and quantified as the ratio of sessile to total cells. Finally, antimicrobial activity was assessed regarding five pathogens (*E. coli*, *L. monocytogenes*, *S. enterica*, *S. aureus*, *C. albicans*) using the double-layer agar method. Hemolytic Activity: To determine the hemolytic profile, overnight yeast cultures grown in YPD broth (1% *w*/*v* yeast extract, 2% *w*/*v* peptone, 2% *w*/*v* glucose) were streak-inoculated onto Columbia agar plates supplemented with 5% (*v*/*v*) defibrinated sheep blood. The plates were incubated at 30 °C for 48 h under aerobic conditions. Evaluation was based on visual inspection of the lysis zones surrounding the colonies: complete red blood cell lysis corresponds to beta-hemolysis, while partial lysis corresponds to alpha-hemolysis, and the complete absence of a lysis zone or discoloration was classified as non-hemolytic gamma-hemolysis, confirming safe status. Gelatinase Activity: Gelatin hydrolysis was assessed by spot-inoculating the yeast isolates onto nutrient agar (0.3% *w*/*v* beef extract, 0.5% *w*/*v* peptone) supplemented with 3.0% (*w*/*v*) gelatin. Following incubation at 30 °C for 48 h, the plates were flooded with a saturated mercuric chloride solution to precipitate intact proteins. The evaluation criteria defined a positive gelatinase result according to the presence of a transparent zone of clearance surrounding the colony (indicating liquefied gelatin), whereas a completely opaque agar medium across the entire plate confirmed the absence of gelatinase activity. DNase Activity: Extracellular deoxyribonuclease production was tested by spot-inoculating the strains onto DNase Test Agar containing 0.2% (*w*/*v*) DNA and 0.005% (*w*/*v*) methyl green indicator. The plates were incubated at 30 °C for 48 h. A positive result was determined by the shifting of the color indicator, yielding a clear halo against the green background around the yeast growth due to DNA degradation, while an uncleared green agar matrix signified a negative (safe) result. All assays were performed in triplicate using three independent biological replicates (n = 3).

### 2.4. Production of “Kombucha-like” Beverage

Micro-fermentation trials were conducted in black tea purchased at a local market (15 g) added to 1000 mL boiling water and left to infuse for 5 min. The obtained tea solution was cooled to room temperature, and the tea leaves were removed by filtration. The solution was supplemented with sucrose (75 g/L).

Yeast strains for the ‘kombucha-like’ trials were screened stepwise to establish two distinct pools: a comprehensive ‘yeast pool’ (YP) featuring 18 biotypes representative of the whole natural kombucha microbiota, and a selective ‘probiotic pool’ (PP). This PP comprised only the three probiotic strains 35-B, 43-G, and 43-Q. They simultaneously outperformed all other isolates in gastric survival (>80%) and bio-adhesion efficiency to Caco-2 cells (up to 99.5%). Four conditions were tested using a yeast pool of 18 isolates and a probiotic pool (PP) of 3 selected strains (two strains of *T. delbrueckii* and one strain of *Z. bailii*), both independently and co-inoculated with *L. thermotolerans* (DiSVA 322) as a complementary strain (YP/L.t and PP/L.t). *L. thermotolerans* was deliberately included to enhance the sensory acidity (via lactic acid production) and mimic the typical tartness of traditional kombucha, which is otherwise lacking when using exclusively the isolated yeast pools without bacteria. Each yeast strain was inoculated at a concentration of 10^6^ cells/mL, except for the *B. anomalus* strains, which were inoculated at 10^4^ cells/mL. In co-inoculated fermentations, *L. thermotolerans* was also added at a final concentration of 10^6^ cells/mL. Fermentation kinetics and bottle refermentation (0.5 g/L sucrose) were monitored at 20 ± 2 °C, as previously reported by [20]. Each trial was conducted in triplicate. Biomass evolution was assessed via viable cell counts and morpho-microscopic characterization on selective media [25]. In this study, the term ‘kombucha-like’ is used to define beverages fermented exclusively with yeast consortia and complementary strains, mirroring the sensory profile of traditional kombucha while operating under a controlled microbial environment.

### 2.5. Analytical and Chemical Characterization

Antioxidant ability and polyphenol production were determined via DPPH radical scavenging assay and Folin–Ciocalteu reagent (GAE equivalents) [16]. The chemical profile was evaluated by monitoring and enzymatically quantifying sugar consumption (K-SUCGL) (Megazyme, Wicklow, Ireland); pH was measured according to official EU methods. Volatile compounds were analyzed by HS-SPME/GC-FID/MS using a DVB/CAR/PDMS fiber and 3-octanol as an internal standard according to Canonico et al. [20]. Briefly, 5 mL of sample (with 1 g NaCl) was spiked with 3-octanol (1.6 mg/L) as an internal standard. Extraction was performed using a DVB/CAR/PDMS fiber for 30 min at 40 °C, followed by desorption into a Supelcowax 10 capillary column (60 m × 0.32 mm × 0.25 μm). Compounds were identified and quantified using external calibration curves for each analyte.

### 2.6. Sensory Evaluation

Sensory analysis was performed by 11 trained evaluators (7 males and 4 females), aged 24–40. They evaluated the samples using a 1–5 intensity scale across various aromatic and structural descriptors. Testing took place in a dedicated room (22–25 °C) under natural light. Kombucha-like samples of 100 mL were served at 12–15 °C in glasses covered with watch glasses to preserve volatile compounds. Data were statistically analyzed to determine the impact of each descriptor on the overall organoleptic quality. Informed consent was obtained from all participants.

### 2.7. Statistical Analysis

The experimental data for the main analytical character of the kombucha-like beverages have been subjected to analysis of variance (ANOVA). Statistica 7, version 7, was used to evaluate the variance. The significant differences were determined using Duncan’s test, and the results were considered significant if the associated *p* values were <0.05.

## 3. Results

### 3.1. Microbial Composition of the SCOBY Biota

A culture-dependent approach using selective or differential media was applied to detect and quantify the microbial community associated with the SCOBYs (Table 1). The data show significant fluctuations between samples. Specifically, SCOBY 1 exhibited the highest microbial concentrations for both yeasts (5.62 log CFU/g) and bacteria (4.68 log CFU/g). Conversely, SCOBY 6 showed a complete absence of detectable yeasts (ND: not detected) and the lowest bacterial count (2.20 log CFU/g). Such variations are consistent with the nature of kombucha, as the microbial diversity and density within a SCOBY are strongly influenced by its geographic origin, the specific fermentation medium used, and environmental factors like temperature and climate [3,6,12]. As reported in Table 1, the relative richness and distribution of yeast species varied significantly across the SCOBY Hotel gradient. The data concerning SCOBY 8 suggest that the exclusive persistence of yeast species such as *B. anomalus*, *P. membranifaciens*, and *M. guilliermondii* is linked to their high resilience. Given that this sample represents the highest culture age, these species have successfully tolerated the prolonged selective pressure characteristic of the kombucha system (e.g., acidification, ethanol accumulation, and nutrient depletion), emerging as the most adapted biotypes for long-term survival. Regarding the relative abundance of yeast species within each specific kombucha sample, significant differences were observed among the SCOBYs. *B. anomalus* showed the highest relative abundance in older matrices, representing the exclusive yeast species detected in SCOBY 4 and SCOBY 5, and dominating SCOBY 8 (88.8% of the isolates). Conversely, *T. delbrueckii* exhibited its maximum relative abundance in the younger and active pellicle, accounting for 70% of the total isolates in SCOBY 1 (p. 5). Biodiversity peaked in SCOBY 3, where the relative abundance was shared among five different species, led by *P. membranifaciens* (41.6%) and *B. anomalus* (33.3%).

### 3.2. Molecular Identification and Strain Genotyping

From an initial pool of 102 isolates obtained from kombucha, 58 representative strains were selected for molecular identification via ITS region sequencing based on their macro- and micro-morphological traits. Out of these 58 sequenced strains, 52 were successfully identified as yeasts, exhibiting a query coverage ≥ 98% and a sequence identity percentage ≥ 99% with reference type strains in the NCBI GenBank database. To act as representative master strains of each identified cluster, 13 accession numbers (PX503675 to PX503687) were assigned upon GenBank deposition. The unidentified yeast strains did not meet quality standards, preventing reliable sequencing and taxonomic assignment. As summarized in Table 1, the yeast community was dominated by *B. anomalus* (33.3%), followed by *P. membranifaciens* (22.9%), *T. delbrueckii* (16.7%), and *Z. bailii* (10.4%), while *M. guilliermondii* was the least frequently detected species (approximately 4% each). SCOBY 3 displayed the highest biodiversity, harboring five different species, whereas SCOBY 6 contained exclusively bacteria. Molecular typing (Figure S2) further revealed significant intra-specific diversity, identifying multiple biotypes: five for *P. membranifaciens*, four for *T. delbrueckii*, three for *Z. bailii*, and two each for *B. anomalus*, *M. guilliermondii*, and *S. bacillaris*. All identified biotypes were subsequently screened for probiotic potential (Table 1).

### 3.3. In Vitro Safety and Probiotic Evaluation of Yeast Isolates

All 18 biotypes were confirmed as safe, testing negative for hemolytic, gelatinase, and DNase activities. In simulated gastric transit (pH 2.0), all strains exhibited remarkable resilience, outperforming the *S. boulardii* control. However, responses to pepsin and bile salts revealed significant variability (Table 2).

Indeed, *S. bacillaris* 36-H, *M. gulliermondii* 50-L, and *B. anomalus* 50-B and 75-B failed to survive gastrointestinal tolerance. Conversely, the other strains successfully bypassed these barriers, showing robust tolerance to low pH, pepsin, and bile salts. Out of these 14 strains, *T. delbrueckii* (35-H, 43-G) and *S. bacillaris* (50-C) showed the highest potential, with active growth exceeding 7.0 log CFU/mL, while *B. anomalus* and *M. guilliermondii* (50-L) proved highly sensitive, failing to survive bile salt exposure (Table 2). Surface properties varied widely among isolates. Strains *Z. bailii* 51-H, *T. delbrueckii* 35-B and 35-H, and *Z. bailii* 43-Q revealed superior auto-aggregation (>94%), though this was not always correlated with hydrophobicity. Specifically, 51-H and 35-B showed 0% hydrophobicity, suggesting that their aggregation is mediated by specific cell-surface molecules rather than non-specific interactions. Regarding antimicrobial activity (Table 3), *Z. bailii* 51-B exhibited the broadest inhibitory spectrum against *L. monocytogenes*, *E. coli*, and *E. fecalis*. Overall, the isolates showed a clear antibacterial trend, while no activity was observed against *C. albicans*.

### 3.4. Adhesion Efficiency to Caco-2 Cells

The Caco-2 cell adhesion assay (Table 2) conducted on the selected strains revealed significant interactions, with values ranging from 94.80% to 99.50%. Notably, *Z. bailii* 43-Q exhibited the highest adhesion efficiency (99.50%). This suggests a high affinity for the Caco-2 apical membrane, potentially outperforming standard benchmarks in terms of bio-adhesion. In comparison, the reference *S. boulardii* CODEX (C+) showed a robust adhesion of 96.60%, validating the reliability of the experimental platform. While *T. delbrueckii* 43-G (95.30%) and 35-B (94.80%) demonstrated slightly lower adhesion rates, they remain within a highly competitive range for intestinal models. The slightly higher variability observed in *T. delbrueckii* 35-B might suggest a greater sensitivity to the micro-environmental conditions of the cell monolayer compared to 43-Q and 43-G.

### 3.5. Physico-Chemical Parameters of Kombucha-like Beverages

Based on probiotic screening, *Z. bailii* 43-Q, *T. delbrueckii* 35-B, and *T. delbrueckii* 43-G were selected to form the targeted probiotic pool (PP) due to their outstanding survival and superior functional traits. These specific strains were chosen because they simultaneously outperformed all other isolates by achieving excellent simulated gastric and gastrointestinal transit survival rates exceeding 80% (Table 2). Furthermore, they demonstrated exceptional bio-adhesion capabilities, yielding Caco-2 apical membrane adhesion efficiencies of up to 99.50% for strain 43-Q, 95.30% for strain 43-G, and 94.80% for strain 35-B, thereby matching or exceeding standard commercial probiotic benchmarks. Regarding surface properties, strains 35-B and 43-Q exhibited superior auto-aggregation profiles (>94% within 24 h), ensuring robust potential for intestinal barrier colonization and competitive pathogen exclusion. Finally, all three strains were confirmed as safe—testing negative for hemolytic, gelatinase, and DNase activities—and showed no mutual antagonism, ensuring a stable, safe, and highly complementary synthetic consortium tailored for plant-based fermentations. The probiotic pool (PP) of the three selected yeasts and the yeast pool (YP) with 18 biotypes were used to set up a different fermentation kombucha-like beverage with and without *L. thermotolerans*. The fermentation kinetics (Figure S1, Supplementary Materials), expressed as CO_2_ production over 16 days, showed a difference in fermentation dynamics depending on the complexity of the yeast consortia. The co-inoculation with *L. thermotolerans* appeared to enhance the overall metabolic activity, resulting in faster fermentation compared to the other trials. This suggests a synergistic effect among the selected probiotic yeasts, where the presence of specific strains can optimize the fermentation performance in a tea-based (kombucha-like) substrate. Comparing the different consortia (Figure S2, Supplementary Materials), the presence of *L. thermotolerans* (*L.T*.) influenced the survival of other species. In the probiotic pool with *L. thermotolerans* (PP/*L.T*.), the overall microbial stability was higher compared to the more complex yeast pool with *L. thermotolerans* (YP/*L.T*.). While *Z. bailii* and *T. delbrueckii* demonstrated excellent fitness throughout the process, species such as *Brettanomyces* and *P. membranifaciens* were progressively outcompeted, especially in the YP/*L.T*. trial, where their populations dropped below the detection limit by day 12. This suggests that the composition of the initial inoculum is a critical factor for maintaining yeast diversity during the fermentation of plant-based (kombucha-like) beverages. The fermentation parameters and chemical profiles of the different kombucha trials are summarized in Table 4. Regarding sugar consumption, residual glucose was not detected (ND) in any of the trials, indicating an efficient metabolic utilization of this carbon source. In contrast, residual sucrose levels varied among the samples; the PP/*L.T*. trial exhibited a significantly higher concentration compared to the YP and YP/*L.T*. trials, suggesting slightly less efficient sucrose hydrolysis or utilization in this specific consortium. The Total Phenolic Content (TPC) and antioxidant capacity showed significant variations depending on the yeast consortium employed. The YP trial displayed the highest TPC, being significantly different from that in trials PP and PP/*L.T*. Interestingly, while trial PP showed the lowest antioxidant activity, the co-inoculation with strain *L. thermotolerans* significantly restored this activity, reaching levels comparable to the YP trial. This synergic effect suggests that strain *L. thermotolerans* plays a crucial role in maintaining the functional properties of the beverage. All samples exhibited typical kombucha pH values, ranging from 3.3 to 3.5. However, significant differences were observed among all trials; the lowest pH values were recorded in trials supplemented with strain *L. thermotolerans*, indicating a more pronounced acidification, likely due to a higher production of organic acids during the fermentation process.

### 3.6. Volatile Organic Compound (VOC) Profiling

The volatile profile (Table 5) varied significantly with the inoculum, revealing a synergistic effect in *L. thermotolerans* and probiotic pool (PP) co-fermentations. *L. thermotolerans* notably enhanced ethyl esters; specifically, ethyl hexanoate (green apple/pineapple) peaked in the PP/*L.T.* group, increasing sevenfold compared to the yeast pool (YP). While ethyl butyrate (strawberry) was highest in the PP alone, its stabilization during co-inoculation suggests complex metabolic interactions. Terpenes (linalool, nerol, and geraniol) also increased in PP/*L.T*. trials, likely due to glucosidase activity releasing bound varietal aromas. Conversely, α-terpineol was higher in the YP trial, suggesting its biotransformation in more complex communities. The most significant enrichment occurred for 2-phenylethanol and phenylethyl acetate (rose/honey), with the PP/*L.T*. combination yielding the highest concentrations. Furthermore, the significantly higher levels of β-damascenone in this group underscore its potential to enhance sensory complexity. Overall, while the YP trial maintained a standard profile, the PP/*L.T*. co-inoculum acted as an effective “bio-flavoring” strategy, producing a superior aromatic bouquet dominated by esters and floral alcohols.

### 3.7. Sensory Profile of Fermented Consortia

**Figure 2:**
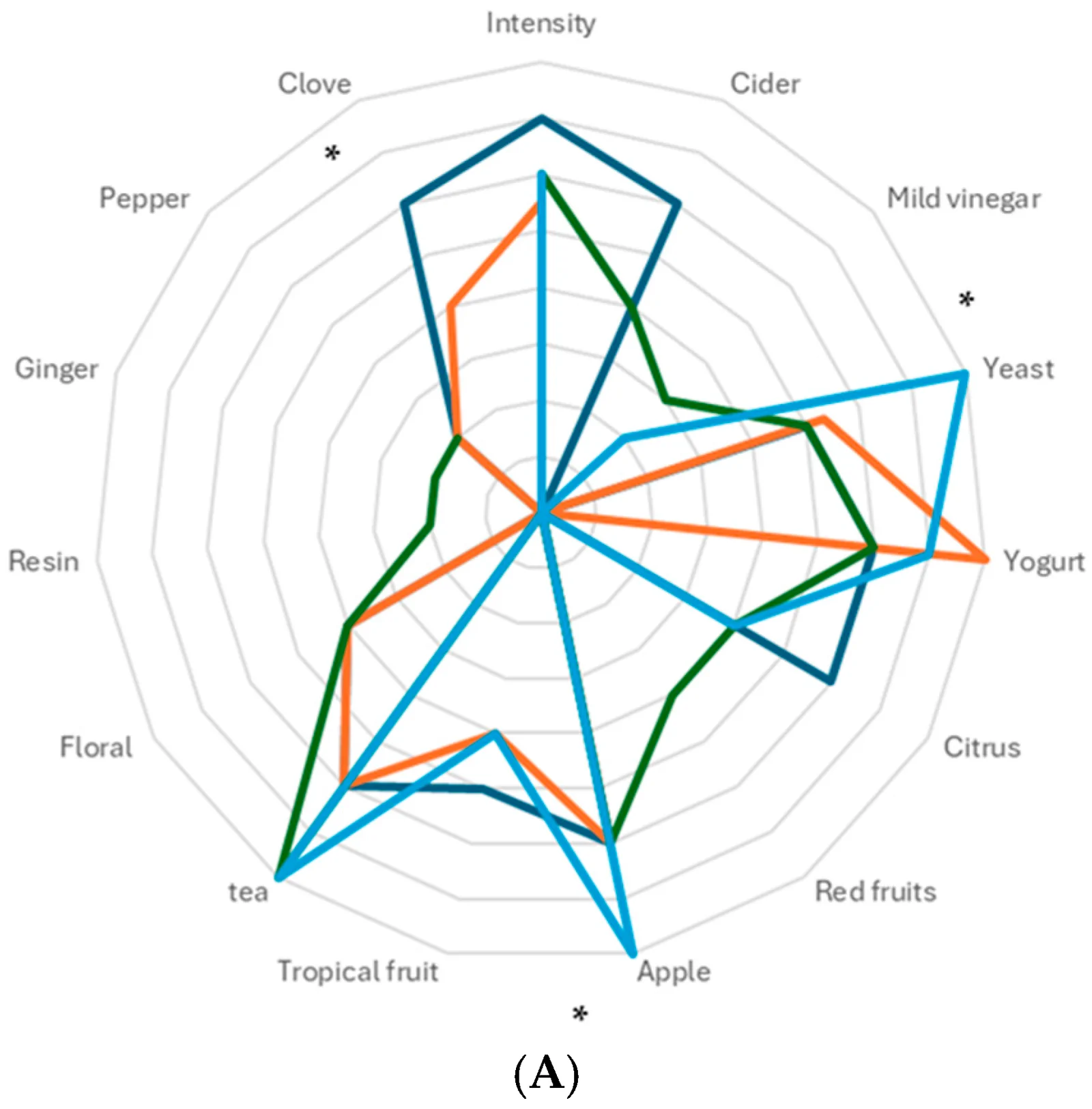

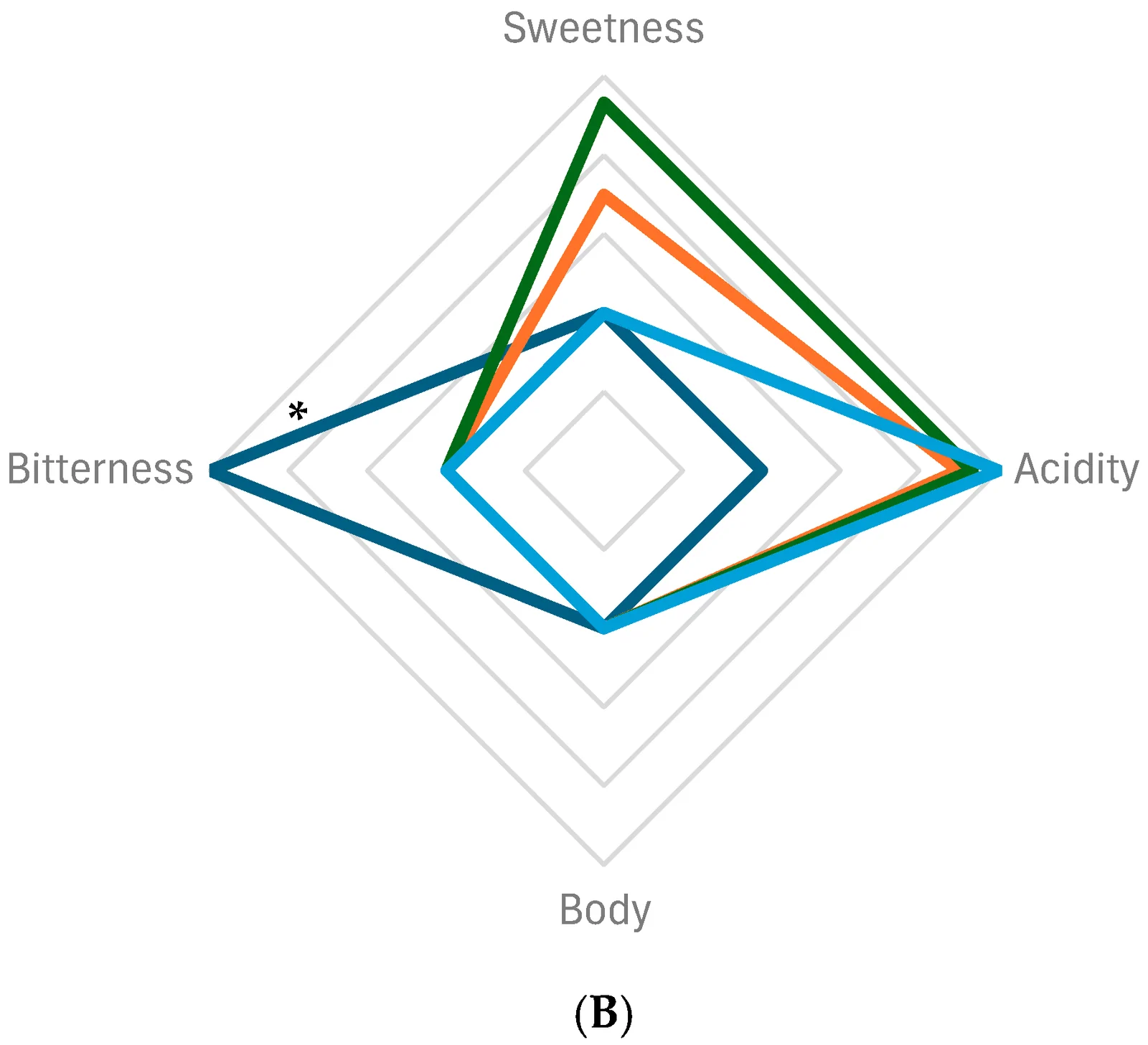
Sensory profile of the kombucha-like beverages fermented with different yeast consortia. Sensory data represent the mean scores awarded by a panel of 11 trained judges. (**A**) Olfactory analysis describing the main aromatic descriptors; (**B**) taste and flavor evaluation of the final beverages. Data represent the mean scores attributed by the sensory panel. PP (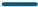); PP/*L.T.* (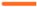); YP (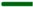); YP/*L.T*. (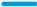). Note: * significantly different (Fisher ANOVA; *p* value 0.05).

The sensory evaluation (Figure 2), visualized through a spider plot, highlighted distinct aromatic notes among the samples. The PP sample stands out for its high aromatic intensity, driven primarily by significantly higher scores in Clove and Mild Vinegar. This suggested a profile leaning towards spicy and pungent notes, potentially linked to a higher concentration of phenolic compounds or a more pronounced organic acid development during processing. In contrast, the YP/*L.T*. sample followed a different sensory profile, characterized by a significantly stronger yeast note and a dominant apple flavor. These results indicated a fresher, more fruit-forward profile, likely influenced by specific fermentation dynamics or the metabolic activity of the starter cultures used. Attributes like “Tea” show high intensity across all variants; the lack of statistical significance suggests that this is a core baseline characteristic of the product rather than a differentiating factor. The use of *L.T.* acted as a powerful modulator for both palatal and aromatic perceptions. In terms of taste, *L.T.* appears to have a “rounding” effect, attenuating the pronounced sweetness in YP and the bitterness in PP samples. Olfactorily, it shifts the profiles toward fermentative and fruity nuances, consistently increasing notes of yeast, yogurt, and apple.

## 4. Discussion

This exploration of kombucha as a reservoir for non-*Saccharomyces* yeasts highlights its potential for developing novel functional beverages. The characterization revealed a complex yeast community dominated by *B. anomalus*, *P. membranifaciens*, *T. delbrueckii*, and *Z. bailii*. This distribution aligns with previous studies identifying these genera as core components of the kombucha SCOBY, well-adapted to its acidic and osmotic environment [13]. Consequently, this natural robustness underscores the safety and resilience of the selected biotypes under simulated gastric conditions, confirming their suitability as probiotics. In particular, the performance of *T. delbrueckii* (35-H, 43-G) and *S. bacillaris* (50-C) in pepsin and bile salts suggests stress-response mechanisms superior to standard commercial controls like *S. boulardii*, a crucial trait for non-*Saccharomyces* candidates to ensure physiological activity and health benefits within the host’s intestinal tract [16,26]. Furthermore, selecting non-*Saccharomyces* yeasts as probiotic candidates provides a distinct therapeutic advantage over conventional bacterial probiotics (e.g., *Lactobacillus* spp.). Due to their eukaryotic nature, these yeast strains are inherently resistant to common antibacterial antibiotics, making them ideal functional vehicles for maintaining intestinal homeostasis and preventing dysbiosis in patients undergoing concomitant antibiotic therapy. The greater survival of *T. delbrueckii* and *S. bacillaris* under acidic stress (pH 2.0) and bile exposure suggests a specialized membrane composition. Non-*Saccharomyces* yeasts often modulate their cell wall chitin and glucan content to maintain integrity against digestive enzymes and bile-induced osmotic shock [27]. Furthermore, the high viability observed suggests that these strains might trigger specific ‘General Stress Response’ (GSR) pathways, involving heat-shock proteins that protect intracellular proteins from denaturation during gastric transit [28].

A key finding was the lack of linear correlation between auto-aggregation and surface hydrophobicity. Strains such as 51-H and 35-B exhibited near-complete aggregation despite 0% hydrophobicity. This indicates that their aggregative behavior is likely mediated by specific cell-surface components, such as exopolysaccharides or specialized proteins, rather than non-specific hydrophobic interactions [27]. Indeed, the role of cell-wall proteins (e.g., adhesins) and secreted polymers in promoting cellular cohesion is well-documented in non-*Saccharomyces* yeasts adapted to competitive environments [29]. Furthermore, the high adhesion efficiency to Caco-2 cells (up to 99.5% for *Z. bailii* 43-Q) reinforces their potential for intestinal persistence and competitive exclusion of pathogens like *E. coli* and *L. monocytogenes*, as observed in our antimicrobial assays. This high bio-adhesion capacity, comparable to or even higher than the standard probiotic benchmarks, is a critical trait for modulating the host immune response and providing a protective barrier against foodborne infections [30].

The exceptional adhesion of *Z. bailii* 43-Q to Caco-2 cells indicates a strong expression of surface adhesins, which are glycoproteins facilitating stable colonization of the intestinal mucus. This bio-adhesive potential is a prerequisite for competitive exclusion, where probiotic yeasts physically occupy binding sites, preventing the attachment of enteropathogens like *E. coli* and *L. monocytogenes* [31]. This mechanism, combined with the production of antimicrobial metabolites or ‘killer toxins’ typical of *Zygosaccharomyces* and *Pichia* genera, forms a dual-layer defense system against foodborne infections [32].

The application of these strains in micro-fermentation trials demonstrated that while the YP and PP can drive fermentation, the presence of *L. thermotolerans* is essential for achieving a balanced analytical profile. The significant drop in pH and the recovery of antioxidant activity in PP co-inoculations suggest a metabolic synergy where *L. thermotolerans* acts as a “complementary strain,” enhancing the functional value of the beverage. This bio-acidification role is consistent with the known ability of *L. thermotolerans* to produce lactic acid, which not only defines the sensory profile but also contributes to the microbial stability and safety of the final product [33,34,35,36].

Unlike conventional kombucha, where acidification relies on the unpredictable growth of AAB that typically drives a sharp pH drop through acetic acid accumulation, the YP and PP fermentations developed in this study lack AAB. Consequently, the acidification process is entirely modulated by the yeast consortia. This explains the controlled and less drastic pH reduction, offering a strategic technological advantage for standardizing the chemical and sensory profiles of yeast-only kombucha-like beverages. In this context, while the application of *L. thermotolerans* allowed controlled bio-acidification through lactic acid production, this specific yeast metabolic pathway effectively replaces the traditional volatile acidity (acetic acid) with a cleaner, more balanced fixed acidity (lactic acid), minimizing the harsh, pungent vinegary notes often associated with consumer rejection of traditional kombucha [34].

The volatile profile analysis confirms that co-inoculation functions as a “bio-flavoring” strategy. The sevenfold increase in ethyl hexanoate and the enrichment of 2-phenyl ethanol (rose aroma) and β-damascenone in the PP/*L.T*. trials are particularly striking. This enhancement is likely due to the superior enzymatic activity (e.g., β-glucosidase) of these specific non-*Saccharomyces* consortia, which are more effective at releasing bound varietal aromas than standard yeast populations [18,37]. Such enzymatic potential is a hallmark of certain *Torulaspora* and *Lachancea* strains, often employed to increase the complexity of fermented plant-based matrices [38,39]. Conversely, the lower levels of α-terpineol in co-fermentations suggest its biotransformation into more complex, odorous derivatives, further contributing to the aromatic bouquet’s depth [40,41].

The sevenfold increase in ethyl hexanoate in the PP/*L.T*. trial highlights a significant metabolic ‘cross-feeding’ phenomenon within this synthetic yeast consortium. While *L. thermotolerans* acts as the primary acidifying engine, providing a steady backbone of lactic acid and ensuring microbial stability against spoilage organisms through rapid acidification [19,32,33,35], the specific non-*Saccharomyces* strains in the probiotic pool (*T. delbrueckii* and *Z. bailii*) likely utilize these organic acids and available alcohols as precursors for active esterification. This synergistic interaction not only enhances fruity and floral notes, such as 2-phenyl ethanol, but also represents a valuable ‘bio-flavoring’ strategy. Moreover, while the base matrix (PP vs. YP) defines the primary identity of the product, ranging from spicy/bitter to floral/sweet, the *L. thermotolerans* treatment harmonizes the gustatory profile and enriches the aromatic complexity. Based on previous fermentation models in the literature, this phenomenon could be explained by a potential metabolic cross-feeding, where the lactic acid backbone typically excreted by *L. thermotolerans* acts as an available precursor for subsequent esterification by specialized non-*Saccharomyces* consortia. While individual organic acids and organic acid esters were not explicitly quantified in this kombucha-like model, such a synergistic pathway represents a plausible mechanism to explain the reduction in palatal sweetness and the simultaneous harmonization of the aromatic framework observed here. This suggests that *L. thermotolerans* DiSVA 322 might be effectively used to modulate perceived acidity and indirectly drive specific aromatic outcomes depending on the starting matrix traits. Ultimately, this ‘consortium-based’ approach represents a strategic tool for standardizing the sensory profile of plant-based beverages, successfully circumventing the production of off-flavors, which represents a common bottleneck when fermenting plant-based matrices with wild or unselected starters [33,38]. In this framework, the experimental outcomes validate the three core hypotheses formulated in advance. Regarding hypothesis (i), the non-*Saccharomyces* kombucha isolates, particularly *T. delbrueckii* and *Z. bailii*, exhibited outstanding gastrointestinal survival and in vitro bio-adhesion rates, comparable or superior to commercial probiotic benchmarks. Concerning hypothesis (ii), micro-fermentation trials demonstrated that a select yeast consortium (YP and PP) possesses the necessary metabolic ability to ferment plant-based matrices without requiring AAB. Finally, touching upon hypothesis (iii), co-inoculation with *L. thermotolerans* DiSVA 322 provided a controlled bio-acidification system, successfully shifting the sensory profile from pungent volatile acidity to a balanced, ester-rich aromatic profile. This combination prevented excessive pH drops by replacing harsh, volatile acidity with lactic acid, while enriching the beverage’s functional features and aromatic complexity (e.g., a sevenfold increase in ethyl hexanoate) through metabolic synergy. Together, these findings confirm all the original assumptions, offering a reproducible system for designing non-dairy probiotic beverages.

## 5. Conclusions

In conclusion, this study validates kombucha as a specialized and sustainable ecological niche for the isolation of probiotic yeasts possessing unique technological and functional traits. Crucially, the successful development of a bacteria-free “kombucha-like” beverage demonstrates that the specific combination of the selected probiotic pool (*T. delbrueckii* and *Z. bailii*) co-inoculated with *L. thermotolerans* strain DiSVA 322 represents a highly promising starter culture for next-generation plant-based functional beverages.

It must be emphasized that these technological outcomes, specifically the controlled bio-acidification via fixed lactic acid production, the attenuation of palatal sweetness, and the multi-fold enrichment of volatile esters and floral alcohols, apply uniquely to the individual metabolic profile of *L. thermotolerans* strain DiSVA 322 rather than reflecting a generalized trait of the species as a whole. This targeted, strain-specific, consortium-based approach highlights a reproducible strategy to standardize the sensory and functional traits of non-dairy formulations without compromising probiotic viability or inducing traditional volatile acidity defects.

## Figures and Tables

**Table 1:** The microbial population detected by viable cell counts in all SCOBYs analyzed and the molecular identification. The results are expressed as log CFU/g of the SCOBY. ND: not detected.

Sample	Bacteria (log CFU/g)	Yeasts (log CFU/g)	Isolated Strains (Code)	Identified Species	Biotypes
SCOBY 1	4.68 ± 0.90	5.62 ± 1.30	35-B, 35-D, 35-F, 35-H 36-A, 36-B, 36-G 35-I, 36-C 36-H	*T. delbrueckii* *B. anomalus* *Unidentified* *S. bacillaris*	35-B, 35-H *T. delbrueckii* 36H *S. bacillaris*
SCOBY 2	4.49 ± 2.63	4.36 ± 1.57	42-A, 42-C, 42-K, 42-N, 42-R, 42-U 43-A, 43-Q 43-F, 43-G, 43-L	*P. membranifaciens* *Z. bailii* *T. delbrueckii*	42-U, 42-C, 42-K *P. mebranifaciens* 43-Q *Z. baiili* 43-G *T. delbrueckii*
SCOBY 3	3.00 ± 0.98	5.53 ± 0.91	48-B, 48-C, 48-F, 48-H, 48-O 48-G, 50-M 50-A, 50-B, 50-P, 51-A 50-C 50-L 51-B, 51-H	*P. membranifaciens* *Unidentified* *B. anomalus* *S. bacillaris* *M. guilliermondii* *Z. bailii*	50-B *B. anomalus* 50-C *S. bacillaris* 50-L *M. guilliermondii* 51-H, 51-B *Z. bailii*
SCOBY 4	4.16 ± 2.02	3.19 ± 0.99	52-A 52-B, 52-L	*Unidentified* *B. anomalus*	
SCOBY 5	4.36 ± 3.34	2.13 ± 0.88	61-A 62-A, 62-B, 62-M	*Z. bailii* *B. anomalus*	
SCOBY 6	2.20 ± 1.70	ND	—	*Only bacteria detected*	
SCOBY 7	5.04 ± 0.36	3.39 ± 1.62	71-A, 71-B, 71-C 72-C, 72-L 72-K	*P. membranifaciens* *B. anomalus* *T. delbrueckii*	71-A, 71-C *P. mebranifaciens* 72-K *T. delbrueckii*
SCOBY 8	2.96 ± 0.95	2.40 ± 0.95	74-A, 74-C, 75-A, 75-B, 75-C, 75-D, 75-E 75-S 74-B	*B. anomalus* *M. guilliermondii* *P. membranifaciens*	75-B *B. anomalus* 75-S *M. guilliermondi*

**Table 2:** The probiotic aptitudes of the 18 biotypes.

CODE	SPECIES	37 °C pH 2.0 (log CFU/mL)	37 °C pH 2 Pepsin 3 g/L (log CFU/mL)	37 °C pH 7 Bile Salts 0.3% (log CFU/mL)	% Auto-Aggregation 24 h	% Hydrophobicity	% Adhesion Caco-2 Cells
36-H	*S. bacillaris*	6.29 ± 0.04 ^a^	NG	NG	-	-	-
50-C	*S. bacillaris*	6.57 ± 0.10 ^a^	7.00 ± 0.07 ^a^	7.14 ± 0.04 ^a^	56.31 ± 0.03	ND	-
43-Q	*Z. bailii*	6.26 ± 0.18 ^a^	6.59 ± 0.04 ^a^	6.69 ±0.12 ^a^	94.69 ± 0.04	16.67 ± 0.87	99.50% ± 0.005
51-H	*Z. bailii*	6.65 ± 0.26 ^a^	6.71 ±0.15 ^a^	7.00 ± 0.04 ^a^	99.28 ± 0.04	ND	-
51-B	*Z. bailii*	6.49 ± 0.10 ^a^	6.72 ± 0.10 ^a^	6.73 ± 0.04 ^a^	80.89 ± 0.02	32.65 ± 1.23	-
75-S	*M. guilliermondii*	6.63 ± 0.07 ^a^	6.95 ± 0.04 ^a^	7.15 ± 0.18 ^a^	76.24 ± 0.05	27.22 ± 1.02	-
50-L	*M. guilliermondii*	6.55 ± 0.26 ^a^	NG	NG	-	-	-
50-B	*B. anomalus*	6.30 ± 0.04 ^a^	NG	NG	-	-	-
75-B	*B. anomalus*	6.24 ± 0.26 ^a^	NG	NG	-	-	-
35-B	*T. delbrueckii*	6.60 ± 0.11 ^a^	7.80 ± 0.14 ^a^	7.23 ± 0.07 ^a^	95.66 ± 0.06	ND	94.80% ± 0.04
72-K	*T. delbrueckii*	6.57 ± 0.07 ^a^	7.00 ± 0.17 ^a^	7.00 ± 0.10 ^a^	87.08 ± 0.02	11.11 ± 1.3	-
43-G	*T. delbrueckii*	6.58 ± 0.12 ^a^	7.05 ± 0.18 ^a^	7.16 ± 0.10 ^a^	85.64 ± 0.03	6.15 ± 0.96	95.30% ± 0.01
35-H	*T. delbrueckii*	6.76 ± 0.07 ^a^	7.15 ± 0.04 ^a^	7.41 ± 0.11 ^a^	94.88 ± 0.02	2.6 ± 0.5	-
42-U	*P. membranifaciens*	6.40 ± 0.18 ^a^	6.63 ± 0.04 ^a^	6.47 ± 0.07 ^a^	75.85 ± 0.07	38.78 ± 1.32	-
42-K	*P. membranifaciens*	6.01 ± 0.18 ^a^	6.53 ± 0.10 ^a^	6.30 ± 0.18 ^a^	38.78 ± 0.07	38.64 ± 1.24	-
71-C	*P. membranifaciens*	6.01 ± 0.04 ^a^	6.52 ± 0.04 ^a^	6.06 ± 0.04 ^a^	87.07 ± 0.07	34.31 ± 1.15	-
42-C	*P. membranifaciens*	6.18 ± 0.13 ^a^	6.51 ± 0.13 ^a^	6.27 ± 0.26 ^a^	13.33 ± 0.5	26.47 ± 1.04	-
71-A	*P. membranifaciens*	6.40 ± 0.26 ^a^	6.49 ± 0.04 ^a^	6.38 ± 0.04 ^a^	23.20 ± 0.02	12.6 ± 1.2	-
Codex	*S. boulardii*	5.88 ± 0.04 ^b^	6.89 ± 0.17 ^a^	6.38 ± 0.10 ^a^	96.17 ± 0.25	33.54 ± 0.17	96.60% ± 0.004

The results of the ability of yeasts to grow/survive at 37 °C, pH 2.0, and in the presence of pepsin or bile salts are reported as log CFU/mL. The initial cell concentration was 5.9 log (CFU/mL). Data are expressed as mean ± SD of three independent biological replicates (n = 3). NG: no growth. ND: not detected. Values displaying different superscript letters (^a,b^) within each column are significantly different according to Duncan’s tests (*p* < 0.05).

**Table 3:** Antimicrobial activity of the yeast isolates against sensitive pathogenic bacteria.

Sample’s Code and Species	Pathogens					
	*Listeria* *monocytogenes*	*Escherichia* *coli*	*Salmonella* *enteriditis*	*Candida* *albicans*	*Staphylococcus* *aureus*	*Enterococcus* *fecalis*
35-B *T. delbrueckii*	-	-	-	-	+	+
43-G *T. delbrueckii*	+	-	-	-	-	-
43-Q *Z. bailii*	-	-	±	-	-	-
35-H *T. delbrueckii*	±	-	-	-	-	±
51-B *Z. bailii*	+	+	±	-	-	+
75-S *M. guilliermondi*	-	-	-	-	-	-
Codex	+	+	+	-	-	-

(-) no antimicrobial acidity; (+) presence of antimicrobial activity; (±) partial antimicrobial activity.

**Table 4:** The main analytical characteristics of the final kombucha.

The Main Fermentation Parameters	YP	YP/*L.T.*	PP	PP/*L.T.*
Residual sucrose g/L	0.06 ± 0.06 ^b^	0.53 ± 0.23 ^b^	ND	2.30 ± 1.72 ^a^
Total phenolic compounds g/L	1.36 ± 0.06 ^a^	1.20 ± 0.006 ^ab^	1.04 ± 0.07 ^b^	1.15 ± 0.23 ^b^
Antioxidant activity (%)	89.68 ± 2.43 ^a^	92.62± 2.53 ^a^	71.48 ± 2.02 ^b^	90.04 ± 3.14 ^a^
pH value	3.56 ± 0.014 ^a^	3.39 ± 0.014 ^c^	3.51 ± 0.03 ^b^	3.33 ± 0.01 ^d^

Data represent the mean ± SD of three independent replicates (n = 3). ND: not detected. The initial composition of the sugars in the kombucha-like beverage was as follows: glucose, 0 g/L; sucrose, 61.80 g/L; total phenolic compounds, 1.26 g/L; antioxidant activity, 24.06%; pH value, 6.09. Values displaying different superscript letters (^a,b,c,d^) within each row are significantly different according to Duncan’s tests (*p* < 0.05).

**Table 5:** Evolution of the main volatile compounds (mg/L) in the mixed fermentation.

Volatile Compounds (mg/L)	YP	YP/*L.T.*	PP	PP/*L.T.*
Ethyl butyrate	ND	0.007 ± 0.001 ^c^	0.043 ± 0.005 ^a^	0.012 ± 0.002 ^b^
Isoamyl acetate	0.260 ± 0.004 ^a^	0.252 ± 0.05 ^a^	0.230 ± 0.04 ^ab^	0.193 ± 0.03 ^b^
Ethyl hexanoate	0.110 ± 0.020 ^c^	0.738 ± 0.015 ^b^	0.325 ± 0.030 ^c^	0.801 ± 0.985 ^a^
Ethyl octanoate	0.002 ± 0.0005 ^c^	0.016 ± 0.003 ^a^	0.002 ± 0.001 ^c^	0.007 ± 0.0 ^b^
Linalool	0.093 ± 0.008 ^c^	0.107 ± 0.005 ^bc^	0.115 ± 0.000 c	0.140 ± 0.0299 ^a^
Diethyl succinate	0.007 ± 0.000 ^b^	0.001 ± 0.000 ^c^	0.002 ± 0.000 ^c^	0.017 ± 0.004 ^a^
α-Terpineol	0.170 ± 0.018 ^a^	0.113 ± 0.024 ^b^	0.090 ± 0.032 ^bc^	0.041 ± 0.001 ^c^
Citronellol	0.010 ± 0.0009 ^b^	0.009 ± 0.002 ^b^	0.034 ± 0.037 ^a^	0.0290 ± 0.004 ^a^
Phenylethyl acetate	0.026 ± 0.004 ^b^	0.0217 ± 0.006 ^b^	0.010 ± 0.002 **^c^**	0.084 ± 0.003 ^a^
Nerol	0.031 ± 0.007 ^b^	0.038 ± 0.011 ^ab^	0.033 ± 0.008 ^b^	0.040 ± 0.003 ^a^
Geraniol	0.008 ± 0.003 ^bc^	0.018 ± 0.009 ^b^	0.005 ± 0.004 ^c^	0.0328 ± 0.046 ^a^
2-Phenylethanol	1.73 ± 0.002 ^b^	1.98 ± 0.014 ^b^	1.43 ± 0.012 ^c^	2.10 ± 0.033 ^a^
β-Damascenone	0.018 ± 0.0002 ^c^	0.018 ± 0.007 ^c^	0.022 ± 0.0006 ^b^	0.038 ± 0.007 ^a^

Data represent the mean ± SD of three independent replicates (n = 3). ND: not detected. Values displaying different superscript letters (^a,b,c^) within each row are significantly different according to Duncan’s tests (*p* < 0.05).

## Data Availability

The original contributions presented in the study are included in the article and Supplementary Materials. Further inquiries can be directed to the corresponding author.

## Supplementary Materials

The following supporting information can be downloaded at: https://www.mdpi.com/article/10.3390/jof12080559/s1, Figure S1: Fermentation kinetics of fermentation s in Kombucha like. YP (); YP/LT (); PP () and PP/LT (). Figure S2: Growth kinetics of mixed fermentation in Kombucha-like. Mixed fermentation of *P. membranifaciens* (), *Z. bailii* (), *S. bacillaris* (), *T. delbrueckii* (), *M. guilliermondii* (), *L. thermotolerans* (), *B. anomalus* ().

## References

[1] Jayabalan R., Malbaša R.V., Lončar E.S., Vitas J.S., Sathishkumar M. (2014). A Review on Kombucha Tea—Microbiology, Composition, Fermentation, Beneficial Effects, Toxicity, and Tea Fungus. Compr. Rev. Food Sci. Food Saf..

[2] Fraiz G.M., Costa M.A., Cardoso R.R., Hébert J.R., Zhao L., Corich V., Giacomini A., Milagro F.I., Barros F.A., Bressan J. (2024). Black Tea Kombucha Consumption: Effect on Cardiometabolic Parameters and Diet Quality of Individuals with and without Obesity. Fermentation.

[3] Bishop P., Pitts E.R., Budner D., Thompson-Witrick K.A. (2022). Kombucha: Biochemical and Microbiological Impacts on the Chemical and Flavor Profile. Food Chem. Adv..

[4] Sanwal N., Bareen M.A., Sahu J.K. (2025). Kombucha Fermentation Enhances Bioactive Profile and Sensory Quality of Sea Buckthorn Leaf Infusion. Biocatal. Agric. Biotechnol..

[5] Laavanya D., Shirkole S., Balasubramanian P. (2021). Current Challenges, Applications and Future Perspectives of SCOBY Cellulose of Kombucha Fermentation. J. Clean. Prod..

[6] Landis E.A., Fogarty E., Edwards J.C., Popa O., Eren A.M., Wolfe B.E. (2022). Microbial Diversity and Interaction Specificity in Kombucha Tea Fermentations. mSystems.

[7] Villarreal-Soto S.A., Bouajila J., Pace M., Leech J., Cotter P.D., Souchard J.-P., Taillandier P., Beaufort S. (2020). Metabolome-Microbiome Signatures in the Fermented Beverage, Kombucha. Int. J. Food Microbiol..

[8] Kaashyap M., Cohen M., Mantri N. (2021). Microbial Diversity and Characteristics of Kombucha as Revealed by Metagenomic and Physicochemical Analysis. Nutrients.

[9] Diez-Ozaeta I., Astiazaran O.J. (2022). Recent Advances in Kombucha Tea: Microbial Consortium, Chemical Parameters, Health Implications and Biocellulose Production. Int. J. Food Microbiol..

[10] Li S., Wang R., Liu R., Wang L., Wang X., Wei J., Yuan Y., Yue T., Cai R., Wang Z. (2024). Exploring the Dynamic Characteristic of Typical Kombucha Induced by Symbiotic Microbiota Succession from Four Chinese Regions: A Comprehensive Analytical Framework. Food Res. Int..

[11] Ogunremi O.R., Agrawal R., Sanni A. (2020). Production and Characterization of Volatile Compounds and Phytase from Potentially Probiotic Yeasts Isolated from Traditional Fermented Cereal Foods in Nigeria. J. Genet. Eng. Biotechnol..

[12] Marsh A.J., O’Sullivan O., Hill C., Ross R.P., Cotter P.D. (2014). Sequence-Based Analysis of the Bacterial and Fungal Compositions of Multiple Kombucha (Tea Fungus) Samples. Food Microbiol..

[13] Laureys D., Britton S.J., De Clippeleer J. (2020). Kombucha Tea Fermentation: A Review. J. Am. Soc. Brew. Chem..

[14] Arevalo-Villena M., Briones-Perez A., Corbo M.R., Sinigaglia M., Bevilacqua A. (2017). Biotechnological Application of Yeasts in Food Science: Starter Cultures, Probiotics and Enzyme Production. J. Appl. Microbiol..

[15] Kokkinomagoulos E., Kandylis P. (2024). Sustainable Exploitation of Wine Lees as Yeast Extract Supplement for Application in Food Industry and Its Effect on the Growth and Fermentative Ability of Lactiplantibacillus Plantarum and Saccharomyces Cerevisiae. Sustainability.

[16] Agarbati A., Canonico L., Gattucci S., Ciani M., Comitini F. (2025). From Pollen to Bee Bread: A Reservoir of Functional Yeasts. Fermentation.

[17] Shruthi B., Adithi G., Deepa N., Divyashree S., Sreenivasa M.Y. (2025). Probiotic and Functional Attributes of Yeasts Isolated from Different Traditional Fermented Foods and Products. Probiotics Antimicro. Prot..

[18] Binati R.L., Salvetti E., Bzducha-Wróbel A., Bašinskienė L., Čižeikienė D., Bolzonella D., Felis G.E. (2021). Non-Conventional Yeasts for Food and Additives Production in a Circular Economy Perspective. FEMS Yeast Res..

[19] Morata A., Loira I., Tesfaye W., Bañuelos M.A., González C., Lepe J.A.S. (2018). Lachancea Thermotolerans Applications in Wine Technology. Fermentation.

[20] Canonico L., Agarbati A., Comitini F., Ciani M. (2023). Unravelling the Potential of Non-Conventional Yeasts and Recycled Brewers Spent Grains (BSG) for Non-Alcoholic and Low Alcohol Beer (NABLAB). LWT.

[21] Stringini M., Comitini F., Taccari M., Ciani M. (2009). Yeast Diversity during Tapping and Fermentation of Palm Wine from Cameroon. Food Microbiol..

[22] Agarbati A., Marini E., Galli E., Canonico L., Ciani M., Comitini F. (2021). Characterization of Wild Yeasts Isolated from Artisan Dairies in the Marche Region, Italy, for Selection of Promising Functional Starters. LWT.

[23] Canonico L., Comitini F., Ciani M. (2015). TdPIR Minisatellite Fingerprinting as a Useful New Tool for Torulaspora Delbrueckii Molecular Typing. Int. J. Food Microbiol..

[24] WHO, FAO (2002). Guidelines for the Evaluation of Probiotics in Food.

[25] Agarbati A., Moretti L., Canonico L., Ciani M., Comitini F. (2024). Agro-Ecosystem of Honeybees as Source for Native Probiotic Yeasts. World J. Microbiol. Biotechnol..

[26] Canonico L., Gattucci S., Moretti L., Agarbati A., Comitini F., Ciani M. (2025). Ethanol Reduction in Montepulciano Wine: Starmerella Bombicola Sequential Fermentation at Pilot Scale Under Aeration Conditions. Foods.

[27] Abdul Manan M. (2025). Progress in Probiotic Science: Prospects of Functional Probiotic-Based Foods and Beverages. Int. J. Food Sci..

[28] Schiavone M., François J.M., Zerbib D., Capp J.-P. (2023). Emerging Relevance of Cell Wall Components from Non-Conventional Yeasts as Functional Ingredients for the Food and Feed Industry. Curr. Res. Food Sci..

[29] Sikora A., Grzesiuk E. (2007). Heat Shock Response in Gastrointestinal Tract. J. Physiol. Pharmacol..

[30] Calabrese F.M., Vincentini O., Filannino P., De Battistis F., Prota V., Latronico R., Ripari V., Polo M., De Angelis M. (2026). Non-Conventional Yeasts with the Potential to Bridge Probiotic and Parabiotic Applications in Foods and Dietary Supplements: Genomics and Human Model Interaction. Appl. Food Res..

[31] Kim H., Haque M.A., Razzak M.A., Jang M.J., Song S., Ku S. (2026). Probiotic Development Strategy Centered on Stability and Regulatory Considerations. Compr. Rev. Food Sci. Food Saf..

[32] Alkalbani N.S., Osaili T.M., Al-Nabulsi A.A., Obaid R.S., Olaimat A.N., Liu S.-Q., Ayyash M.M. (2022). In Vitro Characterization and Identification of Potential Probiotic Yeasts Isolated from Fermented Dairy and Non-Dairy Food Products. J. Fungi.

[33] Comitini F., Canonico L., Agarbati A., Ciani M. (2023). Biocontrol and Probiotic Function of Non-Saccharomyces Yeasts: New Insights in Agri-Food Industry. Microorganisms.

[34] Vaquero C., Escott C., Heras J.M., Carrau F., Morata A. (2022). Co-Inoculations of Lachancea Thermotolerans with Different Hanseniaspora Spp.: Acidification, Aroma, Biocompatibility, and Effects of Nutrients in Wine. Food Res. Int..

[35] Morata A., Loira I., González C., Escott C. (2021). Non-Saccharomyces as Biotools to Control the Production of Off-Flavors in Wines. Molecules.

[36] Gobbi M., Comitini F., Domizio P., Romani C., Lencioni L., Mannazzu I., Ciani M. (2013). *Lachancea thermotolerans* and *Saccharomyces cerevisiae* in Simultaneous and Sequential Co-Fermentation: A Strategy to Enhance Acidity and Improve the Overall Quality of Wine. Food Microbiol..

[37] Canonico L., Moretti L., Agarbati A., Comitini F., Ciani M. (2025). Free and Immobilized Cells of Torulaspora Delbrueckii and Lachancea Thermotolerans in Sparkling Wine: Innovative Application in Secondary Bottle Fermentation. Foods.

[38] Canonico L., Agarbati A., Galli E., Comitini F., Ciani M. (2023). Biocontrol Using Torulaspora Delbrueckii in Sequential Fermentation: New Insights into Low-Sulfite Verdicchio Wines. Foods.

[39] Escribano R., González-Arenzana L., Portu J., Garijo P., López-Alfaro I., López R., Santamaría P., Gutiérrez A.R. (2018). Wine Aromatic Compound Production and Fermentative Behaviour within Different Non-Saccharomyces Species and Clones. J. Appl. Microbiol..

[40] Hashimoto E.H., de Cassia Campos Pena A., da Cunha M.A.A., de Freitas Branco R., de Lima K.P., Couto G.H., Binder Pagnoncelli M.G. (2025). Fermentation-Mediated Sustainable Development and Improvement of Quality of Plant-Based Foods: From Waste to a New Food. Syst. Microbiol. Biomanuf..

[41] Buiatti S., Tat L., Natolino A., Passaghe P. (2023). Biotransformations Performed by Yeasts on Aromatic Compounds Provided by Hop—A Review. Fermentation.

