# Supplementary material for: Kombucha as a Reservoir of Probiotic Yeasts Tailored for Plant-Based Fermentations
Source: J Fungi (Basel). 2026 Jul 30;12(8):559. doi: 10.3390/jof12080559 (PMC13514320; doi:10.3390/jof12080559)
Supplement: Supplementary file 1 [file jof-12-00559-s001.zip › jof-4436707-supplementary.pdf]

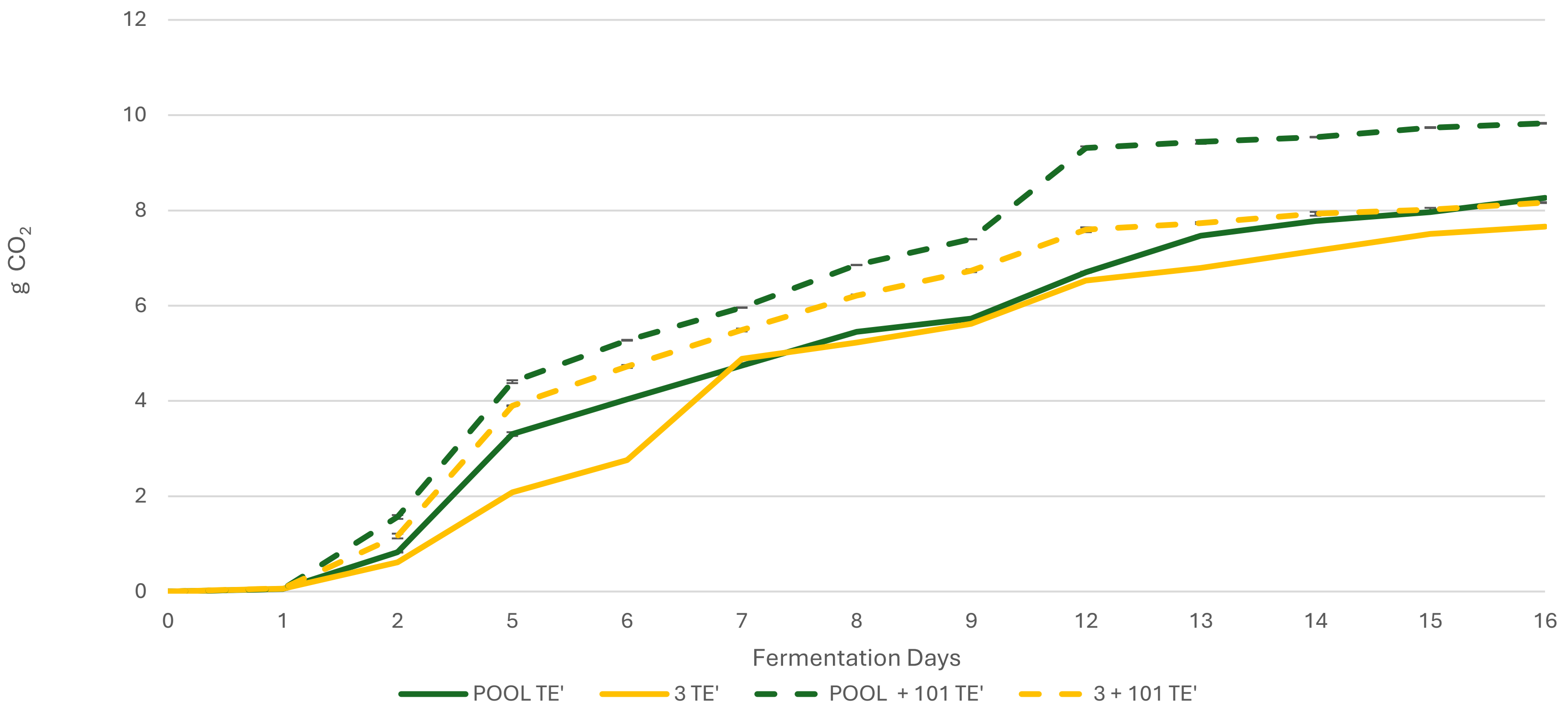

Figure S1. Fermentation kinetics of fermentation s in Kombucha like.YP ( ); YP/LT ( ); PP ( ) and PP/LT ( )

Kombucha

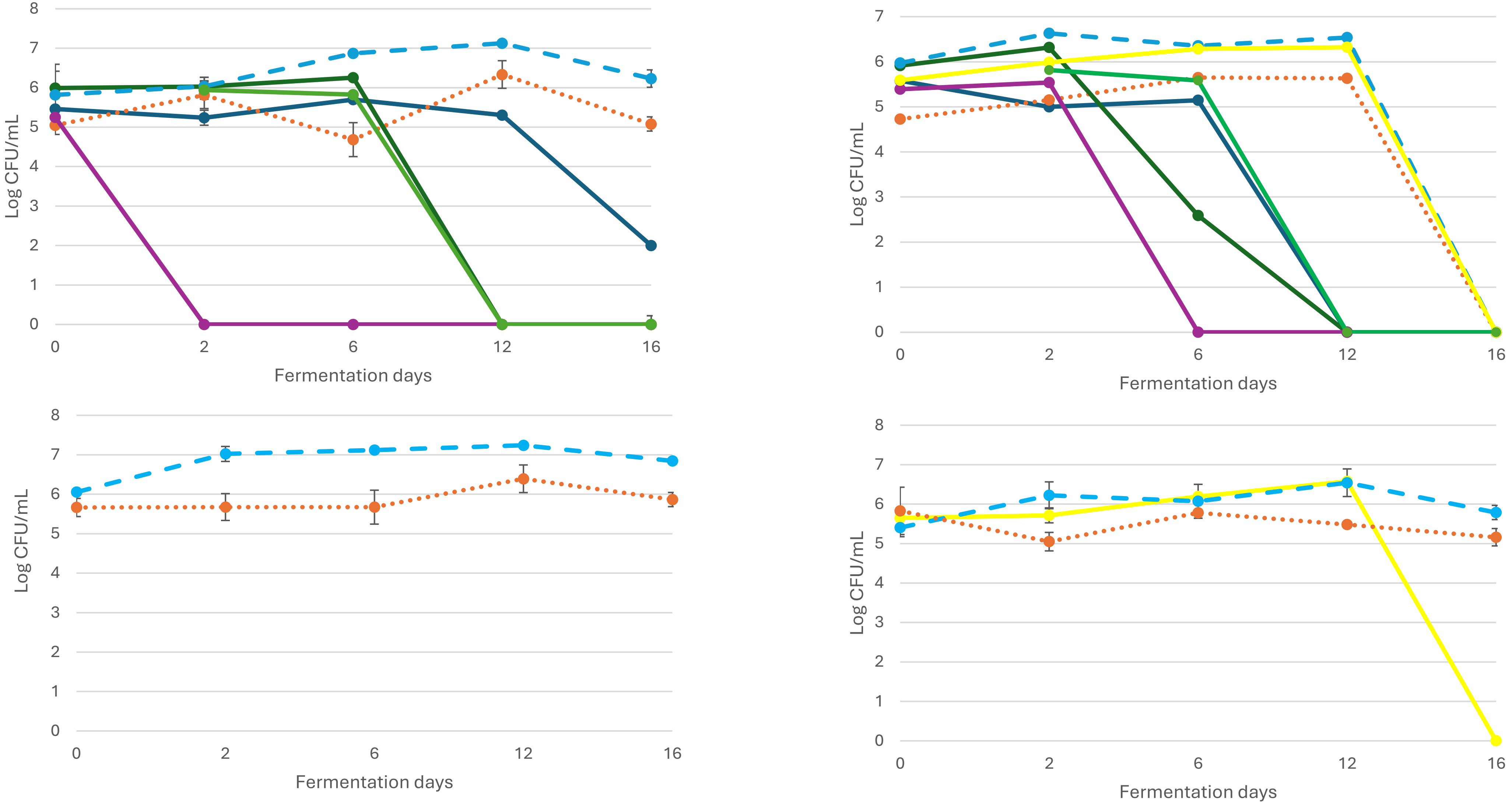

Figure S2. Growth kinetics of mixed fermentation in Kombucha -like
Mixed fermentation of *Pichia membranifaciens* ( ), *Zygosaccharomyces bailii* ( ), *Starmerella bacillaris* ( ) *Torulospora delbrueckii* ( ) *Meyerozyma guilliermondii*.( ) *Lachancea thermotolerans* ( )*Brettanomyces*, ( )
